# Early results after operatively versus non-operatively treated flail chest: a retrospective study focusing on outcome and complications

**DOI:** 10.1007/s00068-018-0961-4

**Published:** 2018-05-21

**Authors:** Mathieu M. E. Wijffels, Tjebbe Hagenaars, Diba Latifi, Esther M. M. Van Lieshout, Michael H. J. Verhofstad

**Affiliations:** Trauma Research Unit, Department of Surgery, Erasmus MC, University Medical Center Rotterdam, P.O. Box 2040, 3000 CA Rotterdam, The Netherlands

**Keywords:** Flail chest, Operative, Outcome, Pneumonia, Rib fracture, Complications

## Abstract

**Purpose:**

Flail chest was traditionally treated non-operatively using mechanical ventilation and pain control. In order to reduce the occurrence of ventilation-associated complications and long-term disability, operative rib fixation is becoming a proven standard therapy for these patients. However, the consequences of the surgical complications may influence success rates negatively. The aim of this study was to compare the outcome of flail chest treatment by surgical rib fixation with non-operative treatment, with special focus on the impact of surgical complications.

**Methods:**

A retrospective case series of operatively treated flail chest patients was compared with non-operatively treated patients. Patients’ injury and treatment characteristics and outcome parameters (e.g., duration of mechanical ventilation, length of Intensive Care stay (ICLOS) and hospital length of stay (HLOS), mortality, surgery-related complications and pneumonia) were collected from the patients’ medical files. Crude and matched-pairs analyses were performed in SPSS.

**Results:**

Twenty-three operatively and 47 non-operatively treated patients were enrolled. Operatively treated patients required significantly shorter mechanical ventilation; median 4 days versus 12 days for the non-operative group (*p* = 0.011). The matched-pairs analysis also showed a lower pneumonia rate (35% versus 80%; *p* = 0.035) and a shorter HLOS (median 21 versus 23 days; *p* = 0.028) in the operative group. No significant differences in duration of ICLOS, and occurrence of other injury-related adverse events were found between both groups.  Seven surgery-related complications occurred, of which three required invasive solutions.

**Conclusions:**

Operative fixation of a flail chest in trauma patients results in a lower rate of pneumonia, less mechanical ventilation days and shorter hospital stay, compared with non-operative treatment, but at the cost of surgery-related complications requiring invasive solutions in some cases.

## Introduction

Chest wall injury is common following blunt trauma [1, 2]. Flail chest is among the most severe chest wall injuries. A flail chest is defined as three or more consecutive rib fractures, in two or more places, creating a flail segment [3, 4]. This is the most severe form of rib fractures and this injury is associated with significant morbidity and mortality rates [5]. Patients suffering from flail chest are traditionally treated non-operatively and require mechanical ventilation and pain control resulting in prolonged stay at the Intensive Care Unit (ICU) [6]. Prolonged ventilation is associated with ventilation-related, potentially lethal complications such as pneumonia, adult respiratory distress syndrome, and sepsis [7, 8]. In addition, long-term morbidities such as pain, disability, respiratory complications, and inability to resume work have been described [9, 10].

Nowadays, a surgical approach for this injury by fixating the ribs is increasingly applied and might offer a decrease in morbidity and mortality rates. Metal plates, intramedullary devices, and encircling wires are implants that are designed to increase chest wall stability supporting the healing process and thereby correction of chest wall deformity [11–14]. More and more evidence shows the benefits of surgical fixation of flail chests over non-operative management [15–18]. Although recent evidence-based guidelines advise surgical fixation in flail chest patients [19, 20], the benefit of rib fixation is not unanimous and needs more comparative studies [21, 22]. However surgical techniques may overcome conservative complications, it may introduce the risk of surgical side-effects. To our knowledge no studies focused extensively on these complications and the required solutions.

In our institution, surgical rib fixation has been a part of the treatment protocol for flail chest since March 2011. This protocol aims to fixate the flail chest operatively within 48 h after hospital admission, to optimize the lung capacity and to reduce the duration of mechanical ventilation, the occurrence of post-traumatic pneumonia and the ICU length of stay.

The aim of this study was to compare the outcome of flail chest treatment by surgical rib fixation with non-operative treatment, with special focus on the impact of surgical complications. Primary outcome measure is the occurrence of pneumonia. Secondary outcome measures included the total hospital length of stay, the duration of mechanical ventilation, surgical complications and the ICU length of stay.

## Patients and methods

### Population

Between March 1, 2011 and March 31, 2014, most patients admitted to a level I trauma center with the diagnosis flail chest have been treated operatively. During this period, surgical rib fixation was the standard-of-care treatment for this injury. Hemodynamically unstable patients or those with a bad or infaust neurological prognosis were treated conservatively for their flail chest, at the consent of the attending surgeon. A historic control group, suffering from flail chest treated non-operatively, at the same hospital between October 1, 2006 and October 31, 2010, was selected. Patients in the control group were identified based upon a text search in the radiology reports and discharge letters. Flail chest was defined as paradoxical movement of the thoracic wall during breathing or three or more adjacent ribs fractured in two or more places as found on the computed tomography (CT). The CT scan made on admission was reviewed by one of investigators (TH) and the research assistant (DL) in order to confirm the diagnosis flail chest and to determine the number of fractured ribs. Patients for whom CT scans were not available, who were transferred to another hospital for treatment, or who died within 24 h after hospital admission were excluded. The medical research ethics committee exempted this study.

### Treatment

Depending on the clinical situation, patients were admitted to the ICU or the clinical ward for monitoring and supportive treatment. Non-operative treatment for patients with flail chest consisted of oxygen support or positive pressure mechanical ventilation for respiratory support, guided by the patients’ oxygen saturation and analgesia for pain control. Individual optimal saturation was determined by the attending ICU-doctor or surgeon. Analgesia included oral medication, intravenous medication, or epidural catheters. Bronchodilators and mucolytics were inhaled at a maximum of six times a day. In the postoperative care, no differences can be found between the surgical and non-surgical treatment protocol.

Operative treatment for patients with flail chest was performed by the attending trauma surgeon. The MatrixRIB (DePuy Synthes, West Chester, Pennsylvania), consisting of pre-contoured metal locking plates, locking screws, and intramedullary splints, was used for the internal fixation of the ribs. A lateral or posterior muscle sparing approach was used. Surgical aim was to stabilize at least one fracture site per rib of the flail segment. Single fractured ribs were not fixated, unless dislocated, as judged by the operating trauma surgeon during the operation. Post-operatively, patients received a chest tube and analgesia for pain control. Postoperative care was provided at the ICU if needed or the clinical ward. Chest tubes were removed when there was no air leak, no pneumothorax on chest radiograph and drainage was less than 100 ml per 24 h.

During the inclusion period selective decontamination of the digestive tract was introduced on the ICU for those patients with an expected duration of mechanical ventilation longer than 48 h.

### Outcome and data collection

The primary outcome, pneumonia, was defined by fever (> 38.5 °C), positive mucus culture, and prescribed antibiotics focused on pulmonary organisms. Secondary outcomes included days of mechanical ventilation, ICU length of stay, hospital length of hospital stay, and other adverse events. Outcome data were collected retrospectively from the patient’s medical files during the first 30 days after trauma. Patient, injury-related, and surgery-related characteristics were collected from the patient’s medical files. Surgical complications were noted in the patient files and were defined as “adverse events in recovery, attributable to the surgery performed for rib fixation”.

### Statistical analysis

Analyses were performed using the Statistical Package for the Social Sciences (SPSS, version 21, SPSS Inc., Chicago, IL, USA). Normality of continuous data was checked using the Shapiro–Wilk test, and homogeneity of variance across groups was determined using the Levene’s test. Baseline characteristics, fracture characteristics, and outcome of patients treated operatively were compared with those of the control group. Continuous data, which were all non-parametric, are presented as medians with first and third quartile. Categorical variables are provided as numbers and percentages. In the crude analysis, data were compared using a Mann–Whitney *U* test (continuous data) or a Chi-squared test (categorical data).

In order to study the effect of operative treatment more specifically, a matched pair analysis was performed. A matched non-operatively treated control was searched for each operatively treated patient. Controls were considered adequate if they were treated non-operatively, had a comparable age (≤ 10 years difference), identical gender, comparable ASA score (American Society of Anesthesiologists classification; ≤1 difference), and a comparable ISS (within the same group of ISS < 25 versus ISS > 25). Controls were selected with replacement; therefor the use of a single control for multiple cases was allowed. Groups were compared using a Wilcoxon signed-rank test (continuous data) or a McNemar test (categorical data). Results with *p* < 0.05 (two-sided test) were regarded as statistically significant.

## Results

### Patient and injury characteristics

During the study period, all 23 operatively treated and 47 non-operatively treated patients were enrolled in the operative and non-operative treatment group, respectively. No data were available on those patients conservatively treated in the “operation period”. Concerning all included patients the median age was 51 years, 52 (74%) were male and 36 (53%) had ASA class I (Table 1). Almost all injuries (*N* = 68; 97%) occurred after a blunt, high energy trauma of which 67 patients (96%) had a unilateral flail segment. Fifty-seven patients (81%) had radiological signs of lung contusion adjacent to the flail chest. ISS score was 32 and 31 in the non-operatively and operatively treated group, respectively (*p* = 0.477).

**Table 1:** Characteristics for the entire study population separated by treatment (*N* = 70)

	Overall (*N* = 70)	Non-operative treatment (*N* = 47)	Operative treatment (*N* = 23)	*P* value
Patients’ characteristics				
Age at trauma (years)^a^	51 (40–66)	49 (40–63)	60 (40–69)	0.268^c^
Male gender^b^	52 (74%)	37 (79%)	15 (65%)	0.354^d^
ASA^b^				
ASA-I	36 (53%)	27 (60%)	9 (39%)	0.171^e^
ASA-II	21 (31%)	13 (29%)	8 (35%)	
ASA-III	11 (16%)	5 (11%)	6 (26%)	
Injury characteristics				
High energy trauma^b^	68 (97%)	45 (96%)	23 (100%)	0.895^d^
Affected side flail segment^b^				
Unilateral	67 (96%)	46 (98%)	21 (91%)	0.499^d^
Bilateral	3 (4%)	1 (2%)	2 (9%)	
Lung contusion^b^	57 (81%)	37 (79%)	20 (87%)	0.628^d^
Unilateral	45 (79%)	30 (81%)	15 (75%)	0.829^d^
Bilateral	12 (21%)	7 (19%)	5 (25%)	
ISS^a^	33 (23–41)	34 (24–41)	29 (20–41)	0.462^c^
GCS on admission^a^	14 (3–15)	14 (3–15)	14 (10–15)	0.502^c^
Intubated on admission^a^	20 (29%)	15 (32%)	5 (22%)	0.554^d^
Total number of fractured ribs^a^	10 (8–12)	9 (8–11)	10 (9–14)	0.130^c^
Fractured ribs at flail segment^a^	5 (4–7)	5 (4–6)	6 (4–7)	0.148^c^
Fractured ribs at non-flail side^a^	5 (3–7)	4 (2–7)	5 (3–7)	0.435^A^

Data are presented as ^a^median (P_25_–P_75_) or as ^b^number (%) and were analyzed with a ^c^Mann–Whitney *U* test, ^d^Fisher’s exact test, or a ^e^Chi-squared analysis

*ASA* American Society of Anesthesiologists, *GCS* Glasgow Coma Scale, *HET* High Energy Trauma, *ISS* Injury Severity Score

The median number of fractured ribs per patient was ten, five at the flail segment and five at the contralateral side. No statistically significant differences were found in baseline characteristics when comparing the two groups.

Of the 23 operatively treated patients, 15 underwent surgical rib fixation within 24 h, an additional 6 between 24 and 48 h and all within 96 h after hospital admission (Table 2). The median duration of anesthesia and surgery was 171 min. A median number of four ribs was fixated. Locking plates were used in 88% of the fixated ribs, and were preferred over intramedullary splints. No intra-operative complications occurred.

**Table 2:** Treatment characteristics for the operative group (*N* = 23)

	Operative treatment (*N* = 23)
Duration of surgery (min)^a^	171 (123–203)
Fixated ribs	4 (4–5)
Rib fixation	
Single fixation	4 (3–5)
Double fixation	0 (0–2)
Plate fixation^b^	12 (52%)
Number of plates^a^	4 (3–5)
Splints^a^	0 (0–1)

Data are presented as ^a^median (P_25_–P_75_) or as ^b^number (%)

### Crude analysis

The rate of pneumonia did not differ statistically significant between the two groups (35% in the operative group versus 57% in the non-operative group; *p* = 0.126; Table 3). When comparing surgically treated patients receiving/not receiving SDD, no differences in the occurrence of pneumoniae were found (respectively, 7/16 versus 1/7, *p* = 0.172). When comparing conservatively treated patients receiving/not receiving SDD, no differences in the occurrence of pneumoniae were found (respectively, 12/24 versus 11/18, *p* = 0.431).

**Table 3:** Outcome for the entire study population separated by treatment (*N* = 70)

	Overall (*N* = 70)	Non-operative treatment (*N* = 47)	Operative treatment (*N* = 23)	*P* value
HLOS (days)^a^	21 (14–32)	23 (14–35)	20 (13–30)	0.495^f^
First IC admission^b^	63 (90%)	42 (89%)	21 (91%)	1.000^g^
Primary ICLOS (days)^a^	7 (3–18)	10 (3–20)	5 (4–11)	0.296^f^
IC readmission^b^	5 (7%)	4 (9%)	1 (4%)	0.932^g^
Secondary ICLOS (days)^a^	4 (3–10)	5 (3–13)	2 (2–2)	0.157^f^
Cumulative ICLOS (days)^a^	8 (3–18)	10 (3–21)	5 (4–11)	0.254^f^
Primary mechanical ventilation^b^	47 (67%)	29 (62%)	18 (78%)	0.264^g^
Primary mechanical ventilation (days)^a,c^	6 (3–14)	**7 (5–17)**	**4 (2–9)**	**0.031**^f^
Re-intubation^b^	5 (7%)	5 (11%)	0 (0%)	0.253^g^
Secondary mechanical ventilation (days)^a^	9 (5–25)	9 (5–25)	NA	NA
Cumulative mechanical ventilation (days)^a^	7 (3–17)	**12 (6–18)**	**4 (2–9)**	**0.011**^f^
Chest tube^b^	62 (89%)	39 (83%)	23 (100%)	0.666^g^
Chest tube (days)^a,d^	7 (5–9)	7 (5–9)	6 (5–9)	0.649^f^
Adverse event^b^	48 (69%)	36 (77%)	12 (52%)	0.075^g^
Surgery-related adverse events				
Surgical site infection^b^	3 (4%)	NA	3 (13%)	NA
Crosslinking ribs^b^	1 (1%)	NA	1 (4%)	NA
Splint through cortex^b^	1 (1%)	NA	1 (4%)	NA
Bleeding^b^	2 (3%)	NA	2 (9%)	NA
General adverse events				
Pneumonia^b^	35 (50%)	27 (57%)	8 (35%)	0.126^g^
Respiratory insufficiency^b^	7 (10%)	6 (13%)	1 (4%)	0.517^g^
Empyema^b^	1 (1%)	1 (2%)	0 (0%)	1.000^g^
Delirium^b^	20 (29%)	17 (36%)	3 (13%)	0.076^g^
Overall in-hospital mortality^b^	6 (9%)	4 (9%)	2 (9%)	1.000^g^
Injury-related in-hospital mortality^b^	2 (33%)	1 (25%)	1 (50%)	1.000^g^

Data are presented as ^a^median (P_25_–P_75_) or as ^b^number (%) and were analyzed with a ^f^Mann–Whitney *U* test, ^g^Fisher’s exact test. Boldface fonts indicate statistically significant differences

Data missing for ^c^three, ^d^four

*ICLOS* intensive care length of stay, *HLOS* hospital length of stay, *NA* not applicable

Most patients were directly admitted to the ICU (89% versus 91%; *p* = 1.000), and five (7%) patients in total were readmitted to the ICU (4 versus 1; *p* = 0.932). Although the median cumulative ICLOS was twice as long in the non-operative group (10 days versus 5 days in the operative group), this did not reach statistical significance (*p* = 0.254).

The percentage of patients requiring mechanical ventilation was similar in both groups (62 versus 78%; *p* = 0.264). Overall, the operative group required shorter mechanical ventilation than the non-operative group (4 days versus 12 days; *p* = 0.011). No statistical differences were found in the number of patients who received SDD when comparing the surgically treated and conservatively treated patients (70% vs. 51%, *p* = 0.162).

The median HLOS was 20 days for the operative group versus 23 days for the non-operatively treated group; *p* = 0.495.

Adverse events occurred in 12 patients (52%) of the operative group versus 36 (77%) in the non-operative group, yet this 25% difference did not reach statistical significance (*p* = 0.075). Overall, six patients died in hospital; two (9%) in the operative group versus four (9%) in the non-operative group (*p* = 1.000). One patient in each group deceased due to the direct effects of flail chest, i.e., pulmonary insufficiency. The other four patients deceased due to traumatic neurological injuries.

Surgery-related adverse events occurred in seven patients (30%). Most common surgery-related complications are the surgical site infections, which present approximately 3 weeks after operation with pus. These abcesses needed to be drained, due to fever and radiological signs of fluid collections without other clear reasons for fever. Additional antibiotics were given in two cases; one with signs of sternal osteitis after a sternotomie for CABG in the past, continuous with the abcess. The other one to prevent from infection of nearby spondylodesis material. The third infected wound was treated successfully with negative wound therapy after drainage.

One of the hardware-related complications was splint-perforation of the visceral facing cortex. In a second case crosslinking two adjacent ribs occurred. In both patients conservative therapy was successful. Two patients showed bleeding out of the surgical wound which has been treated by local wound care. No systematic or (surgical or radiological) interventions were needed.

### Matched-pairs analysis

Of the 23 operated patients, 20 were successfully matched to a non-operatively treated control patient (Table 4). Of the 20 controls, two were randomly picked twice. Baseline characteristics between groups were comparable, except for the median number of fractured ribs at the flail segment; five ribs in the non-operatively treated group versus 6 ribs in the operatively treated group (*p* = 0.021). No significant differences were found comparing conservatively treated patients excluded from matching with operatively or conservatively treated patients included in the matching process.

**Table 4:** Characteristics for the matched pairs separated by treatment (*N* = 40)

	Non-operative treatment (*N* = 20)	Operative treatment (*N* = 20)	*P* value
Patients’ characteristics			
Age at trauma (years)^a^	57 (44–69)	60 (41–69)	0.247^c^
Male gender^b^	15 (75%)	15 (75%)	1.000^d^
ASA^b^			
ASA-I	7 (35%)	8 (40%)	0.126^d^
ASA-II	12 (60%)	7 (35%)	
ASA-III	1 (5%)	5 (25%)	
Injury characteristics			
High energy trauma^b^	20 (100%)	20 (100%)	1.000^d^
Affected side flail segment^b^			
Unilateral	20 (100%)	18 (90%)	0.500^d^
Bilateral	0 (0%)	2 (10%)	
Lung contusion^b^	14 (70%)	18 (90%)	0.289^d^
Unilateral	13 (93%)	13 (72%)	0.500^d^
Bilateral	1 (7%)	5 (28%)	
ISS^a^	32 (21–41)	31 (21–48)	0.477^c^
GCS on admission^a^	13 (3–15)	15 (6–15)	0.243^c^
Intubated on admission^a^	6 (30%)	5 (25%)	1.000^d^
Total number of fractured ribs^a^	10 (8–10)	10 (9–14)	0.097^c^
Fractured ribs at flail segment^a^	5 (3–7)	6 (5–8)	0.021^c^
Fractured ribs at non-flail side^a^	5 (2–7)	5 (3–7)	0.840^c^

Data are presented as ^a^median (P_25_–P_75_) or as ^b^number (%) and were analyzed with a ^c^Wilcoxon signed rank test or a ^d^McNemar test

*ASA* American Society of Anesthesiologists, *GCS* Glasgow Coma Scale, *HET* High Energy Trauma, *ISS* Injury Severity Score

The operatively treated group showed a statistically significantly lower pneumonia rate (35 versus 80%; *p* = 0.035) and a lower median HLOS (21 versus 23 days; *p* = 0.028). (Tables 5, 6) The median number of cumulative ventilation days was lower in the operative group (4 versus 18 days overall; *p* = 0.012). Surgery-related adverse events occurred in six patients (30%).

**Table 5:** Outcome for the matched pairs separated by treatment (*N* = 40)

	Non-operative treatment (*N* = 20)	Operative treatment (*N* = 20)	*P* value
HLOS (days)^a^	**23 (17–42)**	**21 (12–33)**	**0.028**^**c**^
Primary IC admission^b^	19 (95%)	19 (95%)	1.000^d^
Primary ICLOS (days)^a^	12 (3–29)	5 (3–13)	0.131^c^
IC readmission^b^	0 (0%)	1 (5%)	1.000^d^
Secondary ICLOS (days)^a^	17 (17–17)	7 (N.D.)	N.A
Cumulative ICLOS (days)^a^	12 (3–29)	5 (3–13)	0.172^c^
Primary mechanical ventilation^b^	11 (55%)	17 (85%)	0.070^d^
Primary mechanical ventilation (days)^a,e^	**17 (9–22)**	**4 (2–10)**	**0.021**^c^
Re-intubation^b^	2 (80%)	0 (0%)	NA
Secondary mechanical ventilation (days)^a^	25 (N.D.)	ND	NA
Cumulative mechanical ventilation (days)^a^	**18 (12–26)**	**4 (2–10)**	**0.012**^c^
Chest tube (N patients)^a^	15 (75%)	20 (100%)	0.063^d^
Chest tube (days)^a,f^	9 (5–9)	6 (5–9)	0.282^c^
Adverse event^b^	18 (90%)	11 (55%)	0.065^d^
Surgery-related adverse events			
Surgical site infection	NA	2 (10%)	NA
Crosslinking ribs	NA	1 (5%)	NA
Splint through cortex	NA	1 (5%)	NA
Bleeding	NA	2 (10%)	NA
General adverse events			
Pneumonia	**16 (80%)**	**7 (35%)**	**0.035**^**d**^
Respiratory insufficiency	0 (0%)	1 (5%)	1.000^d^
Empyema	0 (0%)	0 (0%)	NA
Delirium	7 (35%)	3 (15%)	0.289^d^
Overall in-hospital mortality^b^	1 (5%)	2 (10%)	1.000^d^
Injury-related in-hospital mortality^b^	0 (0%)	1 (50%)	1.000^d^

Data are presented as ^a^median (P_25_–P_75_) or as ^b^number (%) and were analyzed with a ^c^Wilcoxon signed ranks test or a ^d^McNemar test. Boldface fonts indicate statistically significant differences

Data missing for ^e^two, ^f^three

*CLOS* intensive care length of stay, *HLOS* hospital length of stay, *NA* not applicable

**Table 6:** Surgery-related complications with treatment modality

Surgery-related complication	Presentation (postoperative day)	Treatment	Extra information
Incisional bleeding 1	1st	Local treatment	
Incisional bleeding 2	1st	Local treatment	
Crosslinking ribs	1st	Conservative	On first postoperative X-ray
Splint through cortex	7th	Conservative	On CT due to dyspnea and tachypnea
Surgical site infection 1	20th	Drainage and negative pressure therapy	
Surgical site infection 2	26th	Drainage and antibiotics	Sternal osteitis due to rib fixation after sternotomy in past medical history
Surgical site infection 3	26th	Drainage and antibiotics	

## Discussion

This comparative study showed that operative fixation of a flail chest in trauma patients accounts for a lower rate of pneumonia, less mechanical ventilation days, and shorter hospital stay at the cost of surgery-related adverse events in 30% of the patients.

Rib fractures are common and still have a high morbidity and mortality, dependent on the age and number of fractures, with long-lasting complaints [10, 23]. This stresses the need for new and better treatment modalities. The hypothesis on the beneficial effect of surgical rib fixation is twofold. First, stability of the thoracic wall is improved and as a consequence the residual volume is minimized during spontaneous breathing. This will diminish the pneumonia rate and improve oxygenation resulting in a faster recovery. Furthermore, the indicated pain score after surgical fixation is low enabling productive cough, deep breaths, and movement, protecting from pneumonia and other immobility-related complications [24].

Three randomized controlled trials (RCT) have been published describing only 61 operatively treated patients, compared with 62 non-operatively treated patients [13, 25]. Although based on small numbers of included patients, the results are unanimous; operative fixation of rib fractures reduces the duration of ventilation requirement, lower rate of pneumonia, and shorter ICU and hospital stay. Leinicke *et al*. concluded in a meta-analysis (including two of the three mentioned RCTs), based on nine studies, that operative fixation of flail chest is beneficial in several fields [16]. However, based on 538 patients, only one study in this meta-analysis was rated as high quality. Therefore, case-control studies, such as the current study, are valuable to evaluate daily clinical practice.

Operative fixation of flail chest seems promising but at the cost of surgery-related complications. In our study, 30% of the patients suffered from morbidity directly related to surgery. Granetzny et al. report adverse events directly related to surgery in two of the 20 included patients. Marasco *et al*. and Tanaka *et al*. do not mention the surgical complications [13, 25, 26]. In our series, three out of seven patients needed invasive techniques to solve the complication. This should be taken into account in counselling the patients suitable for rib fixation. Furthermore, it may be valuable to include the number of surgery-related complications in treatment consensus papers, as published by Pierracci (19). Despite this high number of surgery-related complications, the protocol in our hospital did not change based on these adverse numbers. The enthusiasm for splinting decreased over the years and by and large every rib fixation is carried out with plates only. Furthermore, minimizing of the incision, muscle sparing techniques and a low threshold to leave a wound drain postoperatively became more popular.

Sarani *et al*. focusses in their paper on the pitfalls of surgical rib fixation, some of which were found in our case series as well [27]. The larger number of surgical complications in our study may be influenced negatively by definition; crosslinking and splints through the cortex stabilizes the thoracic cage in a not-intended way and was there for scored as a complication in our study. Surgery-related complications of 30% are high, yet without the need of a re-intervention; the impact for the patient is negligible. Fixation of the ribs as soon as the patient is cleared for surgery is favorable with regard to better outcome [11, 28]. Unfortunately, the number of surgically treated patients in our study was too small for a reliable evaluation of surgical timing.

Selective decontamination of the digestive tract is proven to protect from infectious complications like pneumoniae [29] however this effect was not found in our study. Pneumoniae rate was independent on the administration of SDD during ICU stay. Most probably this is due to the small number of patients. The role of SDD in this flail chest population remains unknown since most patients remained on mechanical ventilation longer than 48 h and received SDD in most cases. Larger studies are needed to investigate this role, especially in those patients with concomitant lung contusion.

This study had a number of limitations. First, the short follow-up may have influenced the number of complications. The number of surgical complications may increase after a longer follow-up, since some patients will suffer from symptomatic fixation materials. On the other hand, some non-operatively treated patients will suffer from symptomatic rib non-unions [30]. Second, due to its retrospective character, data are based on medical record information that may be incomplete and inaccurate. Comparison with a historical group may introduce unknown bias due to changes in clinical practice over time. Furthermore, no data were available on the non-operatively treated patients during the “operation-period”. Third, most of the included patients were multi-trauma patients. Therefore, outcome parameters may be influenced by other injuries resulting in less comparable data between groups despite equal ISS score. Fourth, the definition of pneumonia varies widely in rib fixation literature. Therefore, it is hard to compare the outcome of this study with others. Despite these limitations, the scientific evidence favoring surgical stabilization of flail chests has been expanded, with more insight in the accompanying surgical complications.

## Conclusion

From these data, it can be concluded that operative fixation of a flail chest in trauma patients results in a lower rate of pneumonia, less mechanical ventilation days, and shorter hospital stay as compared with non-operative treatment, but at the cost of surgery-related complications.
